# Extracts of *Cistanche deserticola* Can Antagonize Immunosenescence and Extend Life Span in Senescence-Accelerated Mouse Prone 8 (SAM-P8) Mice

**DOI:** 10.1155/2014/601383

**Published:** 2014-01-09

**Authors:** Ke Zhang, Xu Ma, Wenjun He, Haixia Li, Shuyan Han, Yong Jiang, Hounan Wu, Li Han, Tomohiro Ohno, Nobuo Uotsu, Kohji Yamaguchi, Zhizhong Ma, Pengfei Tu

**Affiliations:** ^1^Department of Natural Medicines, School of Pharmaceutical Sciences, Peking University, No. 38 Xueyuan Road, Beijing 100191, China; ^2^Medical and Healthy Analytical Center, Peking University, No. 38 Xueyuan Road, Beijing 100191, China; ^3^Fundamental Research Faculty, Fancl Research Institute, FANCL Corporation, 12-13 Kamishinano, Totsuka-ku, Yokohama, Kanagawa 244-0806, Japan; ^4^Department of Integration of Traditional Chinese and Western Medicine, School of Basic Medical Sciences, Peking University, No. 38 Xueyuan Road, Beijing 100191, China

## Abstract

The senescence accelerated mouse prone 8 substrain (SAM-P8), widely accepted as an animal model for studying aging and antiaging drugs, was used to examine the effects of dietary supplementation with extracts of *Cistanche deserticola* (ECD) which has been used extensively in traditional Chinese medicine because of its perceived ability to promote immune function in the elderly. Eight-month-old male SAM-P8 mice were treated with ECD by daily oral administrations for 4 weeks. The results showed that dietary supplementation of 150 mg/kg and 450 mg/kg of ECD could extend the life span measured by Kaplan-Meier survival analysis in dose-dependent manner. Dietary supplementation of SAM-P8 mice for 4 weeks with 100, 500, and 2500 mg/kg of ECD was shown to result in significant increases in both naive T and natural killer cells in blood and spleen cell populations. In contrast, peripheral memory T cells and proinflammatory cytokine, IL-6 in serum, were substantially decreased in the mice that ingested 100 and 500 mg/kg of ECD daily. Additionally, Sca-1 positive cells, the recognized progenitors of peripheral naive T cells, were restored in parallel. Our results provide clear experimental support for long standing clinical observational studies showing that *Cistanche deserticola* possesses significant effects in extending life span and suggest this is achieved by antagonizing immunosenescence.

## 1. Introduction

*Cistanche deserticola*, one of the most popular traditional Chinese herbal medicines/health products, has been described in a number of historical Chinese herbal pharmacopoeias as having antiaging properties. Consequently it has been widely used in China for treating various age-related disorders including senile dementia, impotence, infertility, chronic infection, and hematopoietic disorders in the elderly [1]. Modern chemical approaches have allowed two principal types of compounds, phenylethanoid glycosides and oligosaccharides, to be isolated as the main active ingredients of *Cistanche deserticola* [1]. In the last decade, *Cistanche deserticola* and its extracts have been studied intensively and shown to be capable of protecting neurons from injury induced by neurotoxins [2], inhibiting carbon tetrachloride induced hepatotoxicity [2], and promoting the recovery of bone marrow cells from Co^60^ induced radiation damage [3]. It has also been shown to have anti-inflammatory, antioxidant, and antiaging effects [4]. However, whether *Cistanche deserticola *can increase life span and what are the underlying molecular mechanisms [3] associated with its antiaging properties has not been rigorously tested.

Immunosenescence, that is, alteration of the immune system with age, forms the background against which increased susceptibility to infections, cancer, neurodegenerative diseases, and autoimmune diseases in the elderly has been noted [5]. Therapeutic interventions, such as caloric restriction [6] and vitamin E supplementation, has been reported to be effective at delaying the progression of immunosenescence and hence reducing the morbidity of some age-related diseases as well as prolonging the life span of both humans and rodents [7, 8]. However, studies in this area are complicated by the fact that aging is associated with the paradox of simultaneous immune deficiency and chronic inflammation [9]. This means that simple stimulation of lymphocyte proliferation or anti-inflammation does not represent ideal therapeutic interventions in dealing with aging and age-related conditions [10]. Consequently in searching for medical interventions capable of preventing or alleviating age-related conditions including infections, cancer, autoimmune diseases, atherosclerosis, and neurodegenerative diseases which are leading causes of death and disability, any repair of immune system defects must be accompanied by an inhibition of inflammatory responses.

The senescence-accelerated mouse [11] is an inbred mouse model, derived from the AKR/J strain, that is widely used in studies of aging. The P8 substrain (SAM-P8) of these mice has a markedly shortened life span when compared to the R1 substrain (SAM-R1), which also shows a slower aging process [12]. In parallel with their premature aging, SAM-P8 mice also exhibit increased neurological senescence, immunosenescence, and age-related hematopoietic deficits which closely mimic typical human aging characteristics [13, 14]. Analysis of the underlying mechanisms responsible for the accelerated aging process and age-related disorders indicates that mitochondrial dysfunction [15], oxidative stress, and increased somatic DNA mutation rate all appear to be involved [16, 17]. This mouse system, with its homogeneous genetic background, therefore, provides an excellent experimental model for studying aging and antiaging therapeutics [12].

This study has focused on investigating whether extracts of *Cistanche deserticola* are able to extend the life span of SAM-P8 mice and reverse their immunosenescence status.

## 2. Materials and Methods

### 2.1. Materials

Fresh *Cistanche deserticola* Y. C. MA was collected from various areas in northwest China including Xinjiang, Neimenggu, and Ningxia autonomous regions. The samples used to prepare the extracts were authenticated by Professor Pengfei Tu, a specialist of pharmacognosy at the Department of Natural Medicines, School of Pharmaceutical Sciences, Peking University. The voucher specimen of *Cistanche deserticola* (number CD-2007-03-08) used had been deposited in the herbarium of the School of Pharmaceutical Sciences, Peking University, China.

### 2.2. Preparation of Extracts of *Cistanche deserticola* (ECD) and HPLC Analysis

Air-dried and sliced *Cistanche deserticola* (2.0 kg) was powdered and extracted twice with 70% ethanol for 1 hour. The ratios of plant/ethanol used were 1/6 (w/w) in the first extraction and 1/4 (w/w) in the second. The two extracts were combined and filtered before being concentrated to a relative density of 1.10–1.15 under the reduced pressure at 60°C. This concentrate was then vacuum-dried and the resulting powder was the extract of *Cistanche deserticola* (ECD) used throughout this study.

The components of ECD were analyzed using HPLC as previously described [1]. Briefly, 100 mg of ECD powder was dissolved in 10.0 mL H_2_O and after filtration injected into the HPLC. HPLC analysis was performed on an Agilent 1100 liquid chromatography system (Agilent Co., USA) The mobile phase consisted of a mixture of methanol (A) and 0.10% methanoic acid (B). A gradient chromatography program was employed; this was 26.5% (A) and 73.5% (B) in 0–7 min, 26.5–29.5% (A) and 73.5–70.5 (B) in 8–10 min, and 29.5% (A) and 70.5% (B) in 20–27 min. The flow rate was held constant at 1.0 mL/min, the injection volume was 10 mL, and the column temperature was maintained at 25°C. A UV detector set at 330 nm was used to monitor the column outflow and generate chromatograms.

Phenylethanoid glycosides and oligosaccharides were identified from their retention times and absorption spectra. Quantification was carried out by external standard calibration curves. The yield of extracts of *Cistanche deserticola* (ECD) was about 3.33% and the content of the phenylethanoids was 17.94%. Acteoside and echinacoside were two major constituents in this fraction, with their contents being 3.80% and 8.25%, respectively. The oligosaccharides account for 82% of total ECD. The concentrations of the main active components of ECD are shown in Table 1.

### 2.3. Analysis of Animal Life Spans

Male SAM-P8 and control SAM-R1 mice were purchased from the Laboratory Animal Breeding and Research Center of Peking University Health Science Centre (Beijing, China). This study was approved by the Peking University Animals Research Committee and carried out according to the guidelines for the Care and Use of Laboratory Animals at Peking University. The certification number of these mice was SCXK2001-2008. The mice were kept in standard metabolic cages in environmentally controlled conditions (22 ± 2°C, 45–60% humidity, and 12 h light/dark cycle) and allowed free access to food and water. After 2 weeks of acclimation, animals were divided into five groups: 3 ECD treatment groups, a SAM-R1 control group, and a no treatment SAM-P8 control group. The diet of the 3 treatment groups was supplemented with diet mixed with different concentrations of ECD: yield of low (50 mg/kg), medium (150 mg/kg), and high (450 mg/kg) average doses of ECD daily. The SAM-R1 and SAM-P8 no treatment control groups were fed with the same diet without ECD. Food intake measurement of all animals was carried out every three days throughout the experiment. Blood pressure and heart rate monitoring were performed noninvasively every three months for the duration of the experiment to determine the health status of the mice. The life span of each mouse was recorded until the death of all animals.

### 2.4. Analysis of Naive T Cells, Memory T Cells, NK Cells, and Sca-1 Positive Cells in Peripheral Blood and Spleen Cell Populations by Flow Cytometry

Eight-month-old male SAM-P8 mice and control SAM-R1 mice were divided into 5 groups: 3 ECD treatment groups of SAM-P8 mice, a SAM-P8 no treatment control group, and a SAM-R1 control group. The diet of the 3 treatment groups was supplemented by daily gavage of ECD at doses of 100 mg/kg, 500 mg/kg, or 2500 mg/kg. After 4 weeks of treatment, the animals were fastened for 12 hours and then anesthetized with pentobarbital sodium solution (50 mg/kg, peritoneal injection, i.p.), and the blood was collected and treated with an anticoagulant, sodium citrate (2 mg/mL). The blood lymphocytes were collected and analyzed.

Then the mice were swabbed with 75% alcohol and abdominal cavities were opened in an ultraclean cabinet (fume hood). The spleens were washed with PBS; then ground and filtrated with a cell strainer (70 *μ*m). The resulting suspension of splenic cells was washed and resuspended in PRMI 1640 medium at a concentration of 2 × 10^6^/mL. The splenic cells from each mouse were aliquoted into 6 tubes for flow cytometry analysis. The final concentration of splenic cell suspension in each tube was 2 × 10^5^/100 *μ*L.

Peripheral blood lymphocytes and splenic lymphocytes were separately incubated with FITC-CD4, Percp-CD3, Percp-CD8a, PC-CD28, PE-CD44, FITC-CD45RB, PE-CD49b/pink, and FITC-ly-6A/E (Sca-1) (all purchased from BD Biosciences, San Diego, CA, USA) monoclonal antibodies at room temperature for 30 min. The lysing solution, which is specifically intended for lysing red blood cells while preserving the leucocytes, thus eliminating the interfering cells for flow cytometric analysis, was incubated with blood in the dark at room temperature for 5 min. After centrifugation at 500 ×g for 5 min, the supernatant was decanted. Then pelleted cells were washed 3 times with PBS, resuspended in ice-cold phosphate buffered saline (PBS, pH 7.4) containing 0.01% sodium azide, and subjected to FCM analysis. Lymphocyte subpopulations were analyzed by flow cytometry using a FACS Calibur flow cytometer (BD Biosciences, San Diego, CA, USA) and BD CellQuest analysis software.

### 2.5. Assessment of Cellular Viability of Peripheral T Lymphocytes by Annexin V-FITC and PI Double Staining and FACS Analysis

Following dietary supplementation of SAM-P8 mice with three doses of ECD for 4 weeks, the animals were fastened for 12 hours and then anesthetized, and blood was collected and analyzed.

Viable apoptotic and necrotic lymphocytes were quantified using the Annexin V-FITC kit (Beijing Biosea Biotechnology Co., Ltd., Beijing, China). The lymphocytes were subjected to double staining with fluorescein isothiocyanate conjugated labeled annexin V (Annexin V-FITC) and propidium iodide (PI). Briefly, lymphocyte suspensions containing approximately 10^6^ cells/mL were washed twice with PBS and suspended in 200 *μ*L of binding buffer (10 mM Hepes/NaOH, pH 7.4, 140 mM NaCl, 2.5 mM CaCl_2_), and 10 *μ*L of Annexin V-FITC was added to the cell suspension. After 15 min of incubation in the dark at room temperature, a further 300 *μ*L of binding buffer and 5 *μ*L PI were added. Immediately following a further 5 min incubation in the dark, the samples of doubly stained lymphocytes were analyzed using flow cytometer, FACS Calibur (BD Biosciences, San Diego, CA, USA).

### 2.6. Determination of Plasma Cytokines by Cytometric Bead Array (CBA) Immunoassay

Plasma cytokine levels were quantified using a cytometric bead array (CBA) assay kit (BD Biosciences, San Diego, CA, USA) capable of simultaneous detection of IFN-*γ*, TNF-*α*, IL-6, IL-2, IL-10, GM-CSF, and IL-3 in a single sample. Therefore, this multiplexed immunoassay enabled detection of immune stimulatory cytokines (IL-2), proinflammatory cytokines (IFN-*γ*, TNF-*α*, and IL-6), inflammatory inhibitory cytokines (IL-10), and hematopoietic cytokines (IL-3 and GM-CSF). Briefly, 50 *μ*L of plasma was mixed with 50 *μ*L of PE-conjugated cytokine capture beads which had been coated with capture antibodies specific for IFN-*γ*, TNF-*α*, IL-6, IL-2, IL-10, GM-CSF, and IL-3 proteins. Following 2 hours of incubation at room temperature, samples were washed within ice-cold phosphate buffered saline (PBS, pH 7.4) containing 0.01% sodium azide, then fixed in 0.5 mL of 1% paraformaldehyde in PBS and kept at 4°C in the dark until analysis, and then analyzed by FACS Calibur flow cytometer (BD Biosciences, San Diego, CA, USA).

### 2.7. Statistical Analysis

Data are shown as mean values ± S.D. Comparisons between different groups were done by one way ANOVA with post hoc test. In the life span study, the data undergone Kaplan-Meier survival analysis, which included use of both the Log-rank (Mantel-Cox) and Gehan-Breslow-Wilcoxon tests. Statistical analysis was conducted using the Statistical Package for Social Sciences for Windows (SPSS, Chicago, IL) and a *P* value of less than 0.05 was considered to be significant.

## 3. Results

### 3.1. Analysis of Potential Active Components in Extracts of *Cistanche deserticola* (ECD)

As shown in Table 1, ECD is composed mainly of two types of compound, phenylethanoid glycosides and oligosaccharides. In the phenylethanoid glycosides, the echinacoside, acteoside, and 8-epiloganic acid have been identified, whilst in the oligosaccharides, only galactitol was identified.

### 3.2. The Impact of *Cistanche deserticola* Extracts on the Average Life Span of SAM-P8 Mice

For this and subsequent studies eight-month-old male SAM-P8 mice were divided into 4 groups. Among them, one group of mice were fed with a normal diet without ECD, the other 3 groups were separately ingested the diets which contain different proportion of ECD. Mice from the SAM-R1 substrain, which have a normal aging process and life span, were used as a control group in all experiments.

**Figure 1:**
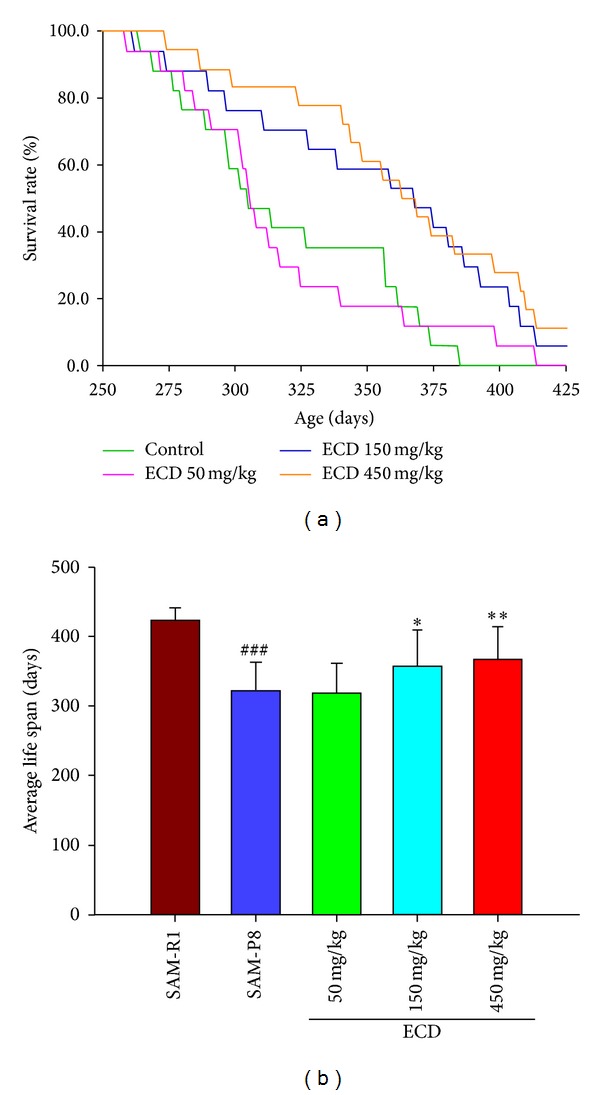
The effects of extracts of *Cistanche deserticola* (ECD) on life span of SAM-P8 mice. Eight-month-old male senescence-accelerated mouse/prone 8 (SAM-P8) mice were randomly divided into 4 groups (in each group, *n* = 17): 3 treatment groups, and a no treatment control group. The nonsenescent substrain (SAM-R1) of mice was used as an experimental control. The food intake of all of animals was monitored throughout the experiment at 3-day intervals. The 3 treatment groups were fed ad libitum on diets supplemented with low (50 mg/kg), medium (150 mg/kg), and high (450 mg/kg) doses of *Cistanche deserticola* extract (ECD). The two control animal groups were fed with the same diet without ECD supplementation. (a) Kaplan-Meier survival curves of SAM-P8 mice dieted ECD or vehicle control. The Kaplan-Meier survival analysis was conducted using the Log-rank (Mantel-Cox) and Gehan-Breslow-Wilcoxon tests. (b) Histogram of the average life span of the groups of mice. The error bars show that the standard deviation from the mean and statistical significance was carried out using ANOVA analysis followed by post hoc *t*-test. ^###^ *P* < 0.001 SAM-P8 versus SAM-R1; **P < 0.01 high dose treated group versus SAM-P8; *P < 0.05 medium dose treated group versus SAM-P8 (in each group, *n* = 17).

Compared to the control SAM-R1 group, the average life span of SAM-P8 mice was significantly shortened (Figure 1(a); *P* < 0.001). Although supplementation of diet with the low dose (50 mg/kg) of ECD failed to produce a significant increase in life span of SAM-P8 mice, at the medium (150 mg/kg) and high (450 mg/mL) supplementary doses there was a dose-dependent increase in life span (Figures 1(a) and 1(b); *P* < 0.05–0.01) that was confirmed by Kaplan-Meier survival analysis, which included use of both the Log-rank (Mantel-Cox) and Gehan-Breslow-Wilcoxon tests.

### 3.3. Reversal of Immunosenescence in SAM-P8 Mice by Extracts of *Cistanche deserticola* (ECD)

**Figure 2:**
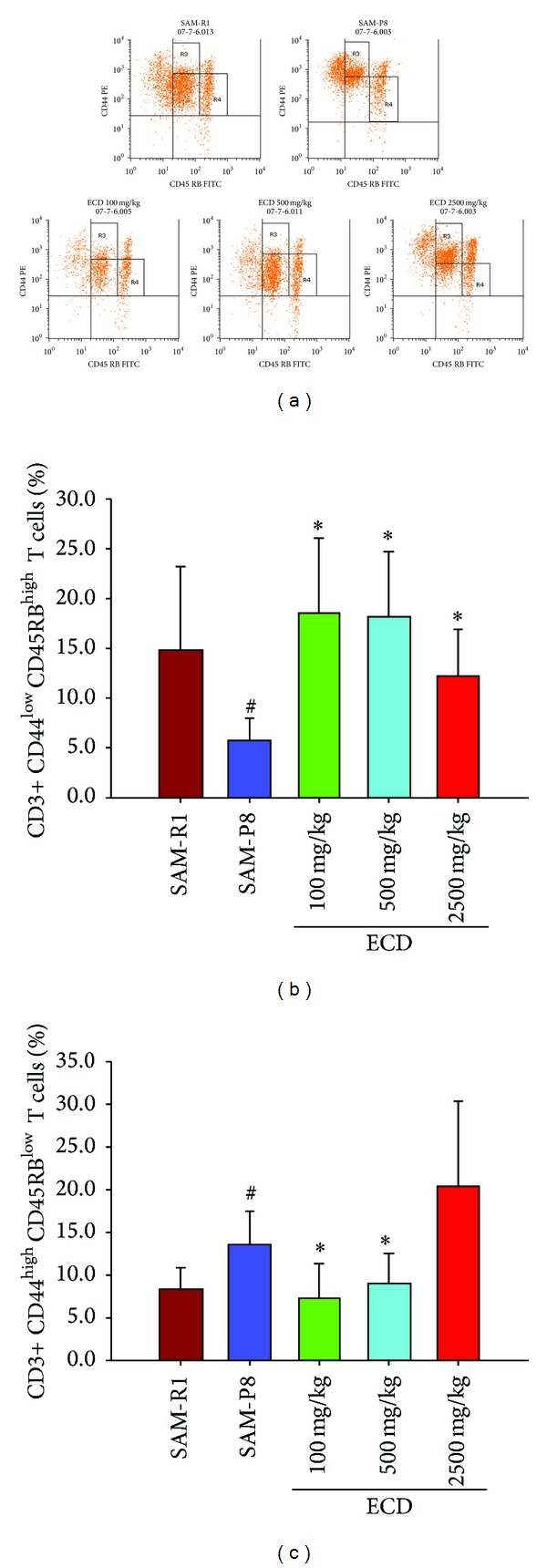
FACS analysis of the effect of dietary supplementation with ECD on naive and memory T lymphocytes in peripheral blood. Eight-month-old male SAM-P8 and control SAM-R1 mice were divided into 5 groups. The 3 treatment groups of SAM-P8 animals were fed for four weeks with diets supplemented with low (100 mg/kg), medium (500 mg/kg), and high (2500 mg/kg) doses of ECD and the two control animal groups were fed on the same diet without supplementation. Following fasting for 12 hours, peripheral blood was collected from anesthetized animals and subjected to FACS analysis all as described in Section 2. (a) Representative FACS plots of the different groups of mice generated by gating on CD3+ T cells. The lines shown on each plot indicate the thresholds used to distinguish CD44^high^ and CD45RB^low^ T cell subsets used to allow identification of naive (CD3+ CD44^low^CD45RB^high^) and memory (CD3+ CD44^high^CD45RB^low^) T cells. (b) Histogram showing the percentage of naive T cells in peripheral blood of the different animal groups. (c) Histogram showing the percentage of memory T cells in peripheral blood of the different animal groups. Bars represent the mean ± SD (in each group, *n* = 10). ^#^ *P* < 0.05 SAM-P8 versus SAM-R1; *P < 0.05 each treated group versus SAM-P8 group (by one way ANOVA with post hoc test).

**Figure 3:**
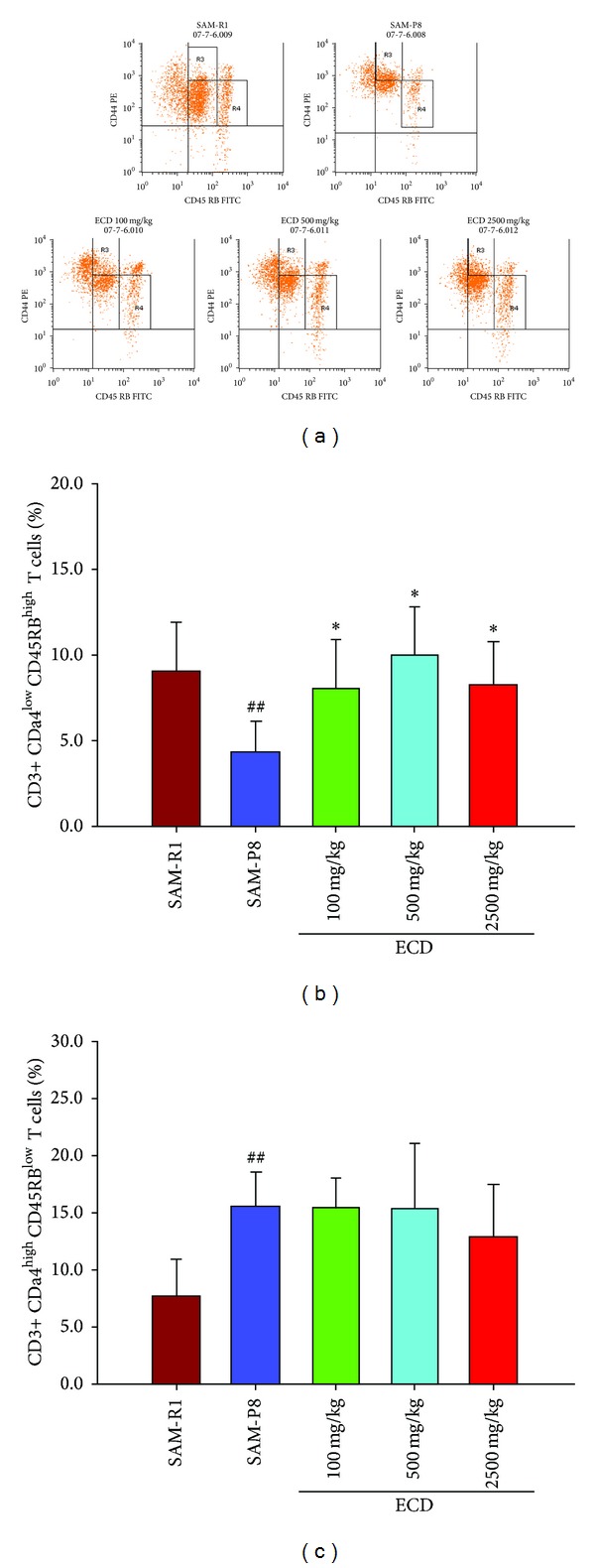
FACS analysis of the effect of dietary supplementation with ECD on naive and memory T lymphocytes in spleen. Eight-month-old male SAM-P8 and control SAM-R1 mice were divided into 5 groups. The 3 treatment groups of SAM-P8 animals were fed for four weeks with diets supplemented with low (100 mg/kg), medium (500 mg/kg), and high (2500 mg/kg) doses of ECD and the two control animal groups were fed on the same diet without supplementation. Following fasting for 12 hours, splenic lymphocytes were collected from anesthetized animals and subjected to FACS analysis all as described in Section 2. (a) Representative FACS plots of the different groups of mice generated by gating on CD3+ T cells. The lines shown on each plot indicate the thresholds used to distinguish CD44^high^ and CD45RB^low^ T cell subsets used to allow identification of naive (CD3+ CD44^low^CD45RB^high^) and memory (CD3+ CD44^high^CD45RB^low^) T cells. (b) Histogram showing the percentage of naive T cells in splenic lymphocytes of the different animal groups. (c) Histogram showing the percentage of memory T cells in splenic lymphocytes of the different animal groups. Bars represent the mean ± SD (in each group, *n* = 10). ^##^ *P* < 0.01 SAM-P8 versus SAM-R1; *P < 0.05 each treated group versus SAM-P8 group (by one way ANOVA with post hoc test).

**Figure 4:**
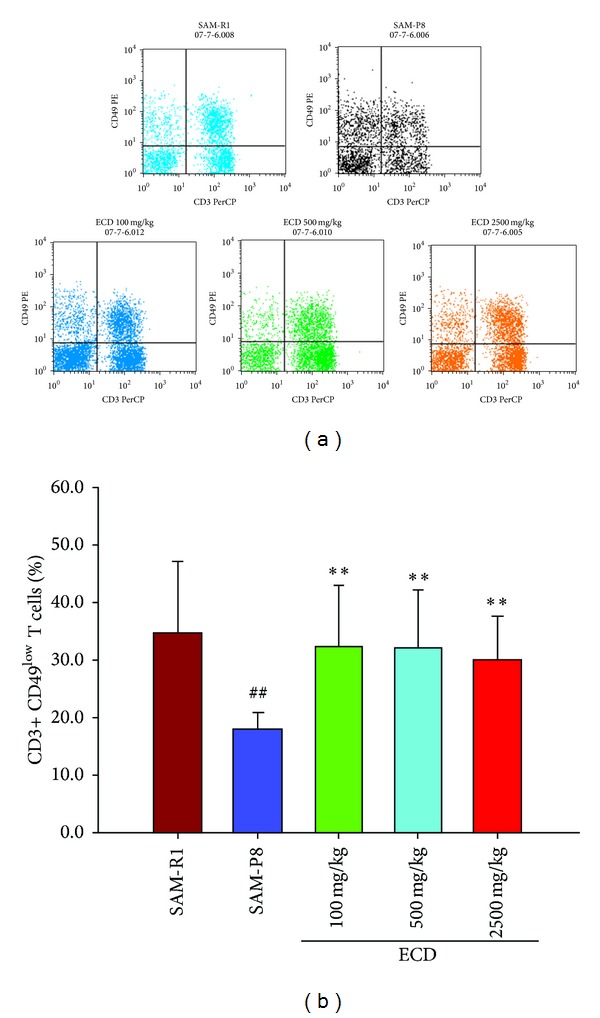
The effects of ECD dietary supplementation on natural killer cells (NK cells, CD3+ CD49+ T cells) in the peripheral blood lymphocytes populations of SAM-P8 mice. Eight-month-old male SAM-P8 and control SAM-R1 mice were divided into 5 groups. The 3 treatment groups of SAM-P8 animals were fed for four weeks with diets supplemented with low (100 mg/kg), medium (500 mg/kg), and high (2500 mg/kg) doses of ECD and the two control animal groups were fed on the same diet without supplementation. Following fasting for 12 hours, peripheral blood was collected from anesthetized animals and subjected to FACS analysis all as described in Section 2. (a) Representative FACS plots of the different groups of mice generated by gating on CD3+CD49+ T cells. (b) Histogram showing the percentage of natural killer T cells in peripheral blood of the different animal groups. Bars represent the mean ± SD (in each group, *n* = 10). ^##^ *P* < 0.01 SAM-P8 versus SAM-R1; **P < 0.01 each treated group versus SAM-P8 group (by one way ANOVA with post hoc test).

**Figure 5:**
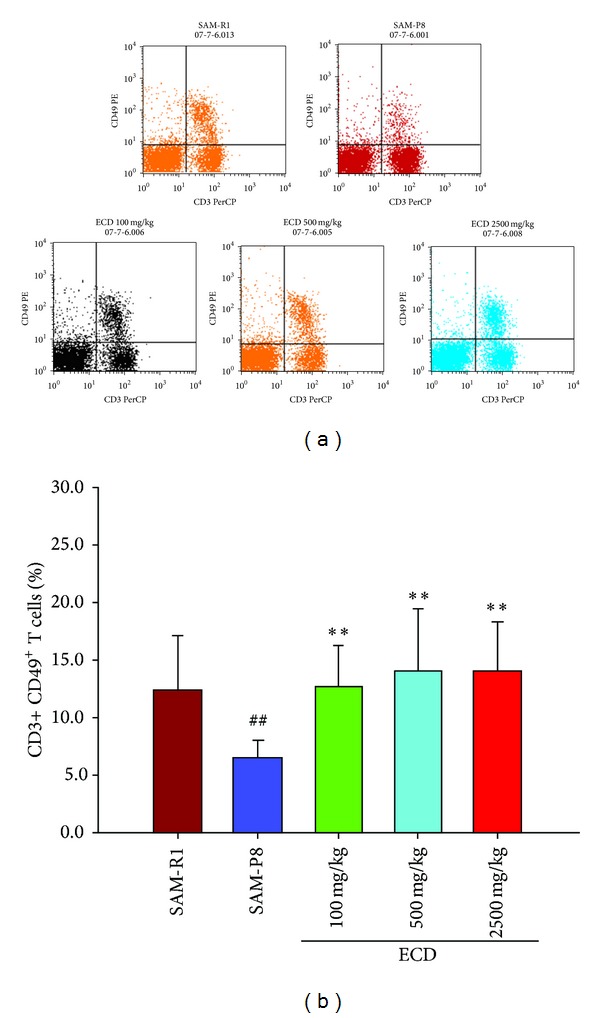
The effects of ECD dietary supplementation on natural killer cells (NK cells, CD3+ CD49+ T cells) in the splenic lymphocytes of SAM-P8 mice. Eight-month-old male SAM-P8 and control SAM-R1 mice were divided into 5 groups. The 3 treatment groups of SAM-P8 animals were fed for four weeks with diets supplemented with low (100 mg/kg), medium (500 mg/kg), and high (2500 mg/kg) doses of ECD and the two control animal groups were fed on the same diet without supplementation. Following fasting for 12 hours, splenic lymphocytes were collected from anesthetized animals and subjected to FACS analysis all as described in Section 2. (a) Representative FACS plots of the different groups of mice generated by gating on CD3+ CD49+ T cells. (b) Histogram showing the percentage of natural killer T cells in splenic lymphocytes of the different animal groups. Bars represent the mean ± SD (in each group, *n* = 10). ^##^ *P* < 0.01 SAM-P8 versus SAM-R1; **P < 0.01 each treated group versus SAM-P8 group (by one way ANOVA with post hoc test).

A decrease of peripheral naive T lymphocytes and concomitant increase of peripheral memory T lymphocytes are prominent features of immunosenescence which are widely regarded as the main underlying reasons for age-related immunological abnormalities. This immunosenescence in SAM-P8 relative to SAM-R1 animals was clearly evident when FACS analysis was used to enumerate (CD3+ CD44^low^CD45RB^high^) lymphocytes as an indicator of naive T cells and (CD3+ CD44^high^CD45BR^low^) lymphocytes as an indicator of memory T cells. Thus, in both peripheral blood (Figure 2) and spleen cell (Figure 3) populations reduced levels of naive T cells and increased levels of memory T cells were seen in SAM-P8 mice. Supplementation of the diet of SAM-P8 mice with ECD was found to be able to reverse these indicators of immunosenescence in a dose-dependent fashion in both peripheral blood (Figure 2) and spleen cell (Figure 3) populations. As an additional indicator of the reversal of immunosenescence by ECD supplementation of diet, the level of natural killer [18] cells, a major cellular marker of the innate immune system, was analyzed. This showed that ECD diet supplementation resulted in a dose-dependent increase in NK (CD3+ CD49+) cells in both peripheral blood (Figure 4) and spleen cell (Figure 5) lymphocyte populations.

### 3.4. Extracts of *Cistanche deserticola* Strengthen the Relative Fluorescence Intensity of Sca-1 Positive Cells in SAM-P8 Mice

**Figure 6:**
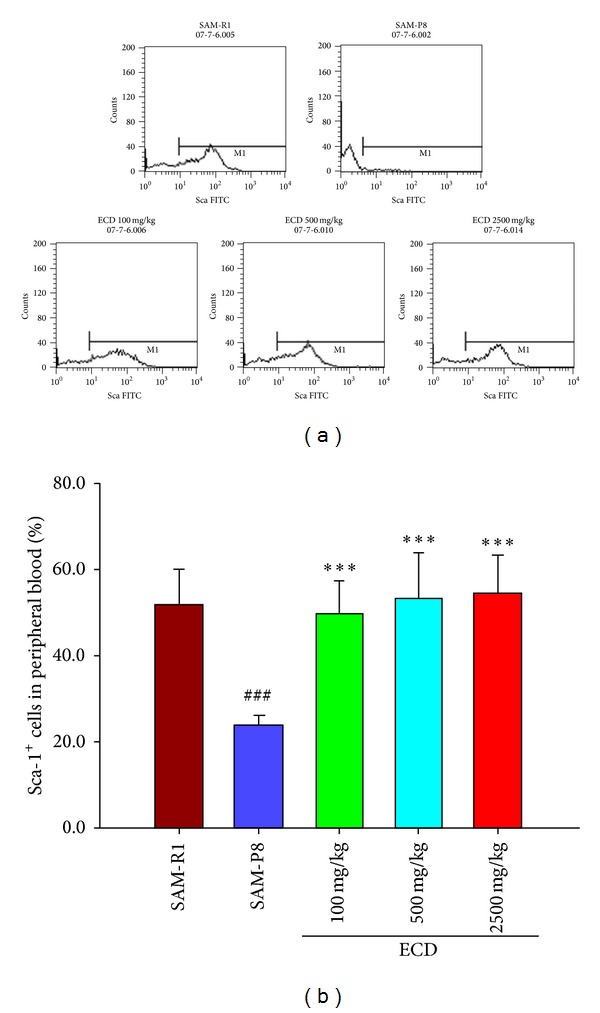
The effects of ECD dietary supplementation on stem cell antigen-1 (Sca-1) in the peripheral blood lymphocytes of SAM-P8 mice. Eight-month-old male SAM-P8 and control SAM-R1 mice were divided into 5 groups. The 3 treatment groups of SAM-P8 animals were fed for four weeks with diets supplemented with low (100 mg/kg), medium (500 mg/kg), and high (2500 mg/kg) doses of ECD and the two control animal groups were fed on the same diet without supplementation. Following fasting for 12 hours, peripheral blood was collected from anesthetized animals and subjected to FACS analysis all as described in Section 2. (a) Representative FACS plots of the different groups of mice generated by gating on stem cell antigen-1 (Sca-1) positive cells. (b) Histogram showing the percentage of stem cell antigen-1 (Sca-1) positive cells in peripheral blood of the different animal groups. Bars represent the mean ± SD (in each group, *n* = 10). ^###^ *P* < 0.01 SAM-P8 versus SAM-R1; ***P < 0.01 each treated group versus SAM-P8 group (by one way ANOVA with post hoc test).

Stem cell antigen-1 (Sca-1) is one of most prominent biomarkers of hematopoietic stem cells (HSC) in bone marrow cell populations. Sca-1 positive cells also represent lymphocyte progenitors that have been newly exported from bone marrow into peripheral blood where they undergo further differentiation into various types of mature lymphocytes. Consequently, these Sca-1 positive cells represent the main source of naive T lymphocytes in peripheral blood.  Figure 6 shows that compared with SAM-R1 control mice, the relative fluorescence intensity of Sca-1 positive cells in SAM-P8 mice was substantially lower. Diet supplementation of SAM-P8 mice with three different doses of ECD was shown to significantly enhance the relative fluorescence intensity of Sca-1 positive cells (Figure 6).

### 3.5. Extracts of *Cistanche deserticola* (ECD) Promote Apoptosis and Inhibit Necrosis of Lymphocytes in SAM-P8 Mice

**Figure 7:**
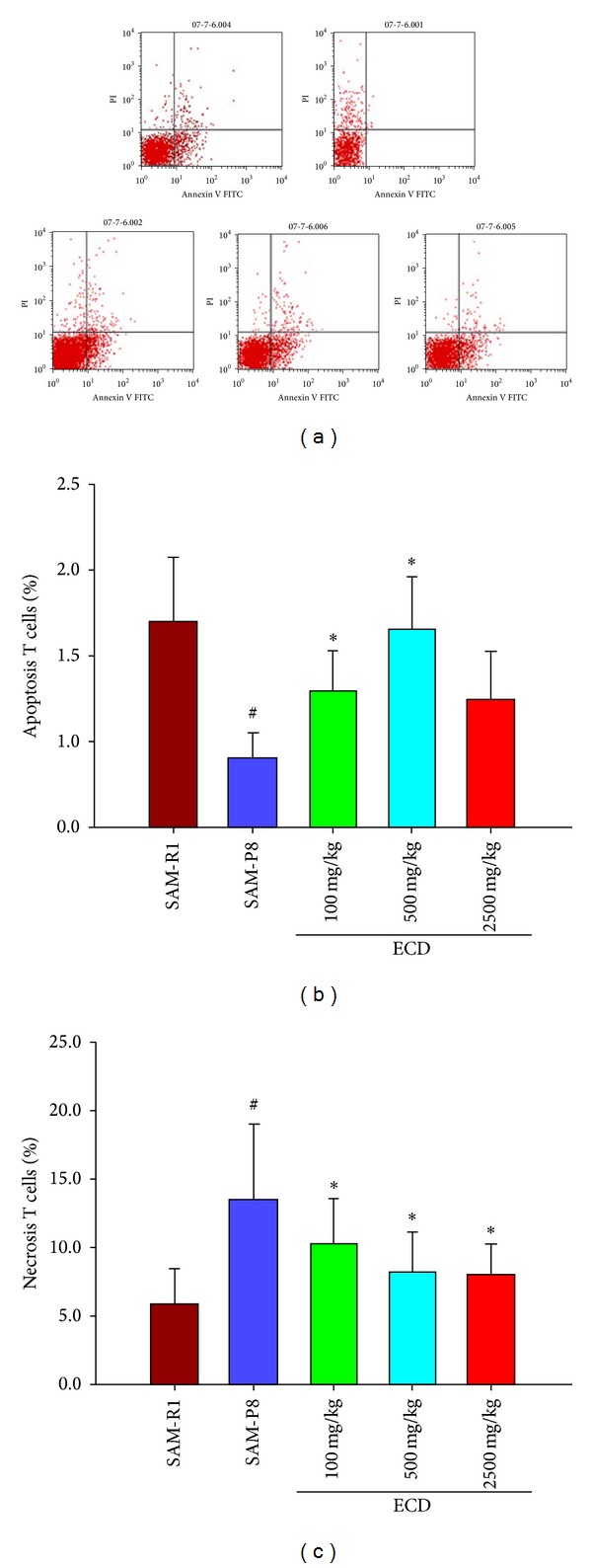
The effects of ECD dietary supplementation on apoptosis and necrosis of lymphocytes in aged SAM-P8 mice. Eight-month-old male SAM-P8 and control SAM-R1 mice were divided into 5 groups. The 3 treatment groups of SAM-P8 animals were fed for four weeks with diets supplemented with low (100 mg/kg), medium (500 mg/kg), and high (2500 mg/kg) doses of ECD and the two control animal groups were fed on the same diet without supplementation. Following fasting for 12 hours, peripheral blood was collected from anesthetized animals and subjected to FACS analysis all as described in Section 2. (a) Representative FACS plots of the different groups of mice generated by gating on double stained blood lymphocytes with Annexin V-FITC/PI. (b) Histogram showing the percentage of apoptotic lymphocytes (Annexin V-FITC positive cells) in peripheral blood of the different animal groups. (c) Histogram showing the percentage of necrotic lymphocytes (PI positive cells) in peripheral blood of the different animal groups. Bars represent the mean ± SD (in each group, *n* = 10).^#^ *P* < 0.05 SAM-P8 versus SAM-R1; ^#^ *P* < 0.05 each treated group versus SAM-P8 group (by one way ANOVA with post hoc test).

The levels of necrosis and apoptosis in blood lymphocyte populations were analyzed by double staining with Annexin V-FITC V/PI. This revealed that the proportion of necrotic lymphocytes in SAM-P8 mice was significantly higher than in SAM-R1 control animals, while the proportion of apoptotic lymphocytes was lower (Figure 7). Supplementation of the diet of SAM-P8 mice with three different doses of ECD was able to inhibit the levels of necrosis seen in peripheral lymphocyte populations, whilst only the two higher levels of ECD supplementation produced a significant change in the level of apoptotic lymphocytes (Figure 7).

### 3.6. Extracts of *Cistanche deserticola* (ECD) Decrease the Proinflammatory Cytokine IL-6 in SAM-P8 Mice

**Figure 8:**
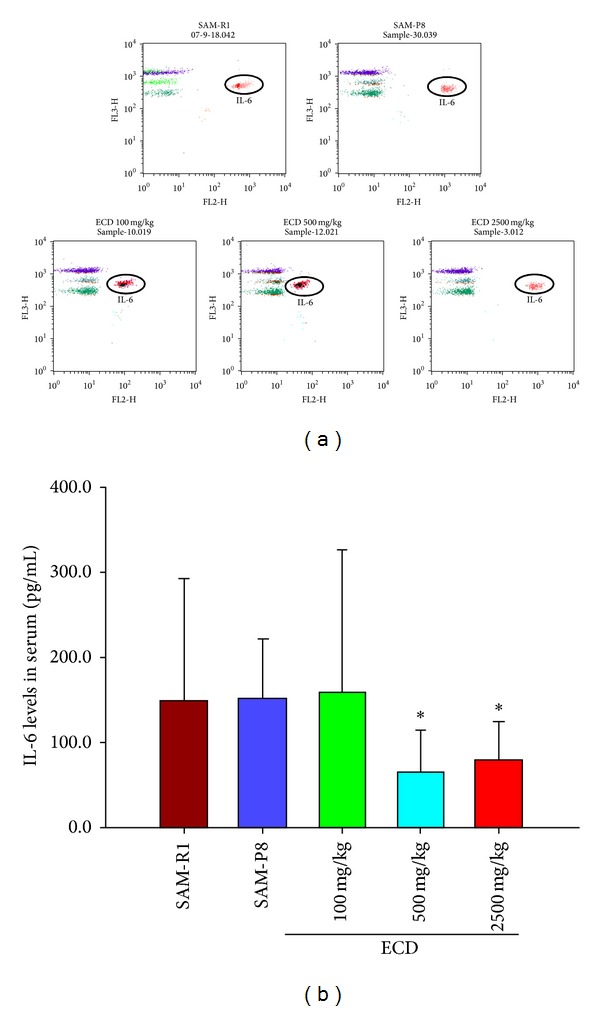
Effects of the ECD dietary supplementation on serum proinflammatory cytokine IL-6 level of SAM-P8 mice. Eight-month-old male SAM-P8 and control SAM-R1 mice were divided into 5 groups. The 3 treatment groups of SAM-P8 animals were fed for four weeks with diets supplemented with low (100 mg/kg), medium (500 mg/kg), and high (2500 mg/kg) doses of ECD and the two control animal groups were fed on the same diet without supplementation. Following fasting for 12 hours, the plasmas were collected and plasma cytokines in the different animal groups were determined by cytometric bead array immunoassay all as described in Section 2. (a) Representative FACS plots of the different groups of mice generated by gating on IL-6 cytometric bead array. (b) Histogram showing the concentrations of cytokine IL-6 in plasma of the different animal groups. Bars represent the mean ± SD (in each group, *n* = 10). *P < 0.001 each treated group versus SAM-P8 group (by one way ANOVA with post hoc test).

As a further indicator of ECD's capacity to influence age-related changes in the immune system, the effect of ECD diet supplementation on the level of a number of cytokines (IFN-*γ*, TNF-*α*, IL-2, IL-6, IL-10, GM-CSF, and IL-3) was analyzed using cytometric bead array analysis (CBA) which involves staining with fluorescent-dye-labeled antibodies coupled with capture cytometric beads. The results (Figure 8) showed that the inflammatory cytokine IL-6 was not increased in 8-month-old SAM-P8 mice compared with control SAM-R1 mice of the same age, but it was significantly increased when compared with 6-month-old SAM-R1 mice (results not shown). However, dietary supplementation with high and medium doses of ECD was able to produce a statistically significant decrease in plasma IL-6 level (Figures 8(a) and 8(b), *P* < 0.05). The plasma levels of the other cytokines examined (IFN-*γ*, TNF-*α*, IL-2, IL-10, GM-CSF, and IL-3) showed no significant variations between SAM-P8, SAM-R1, and ECD treated groups of mice (data not shown).

## 4. Discussion

The immune defense against newly invasive microorganisms or endogenous tumor cells depends on the diversity of T cell repertoire, which in turn relies on the generation and maintenance of naive T cells [19]. During the aging process, the diversity of the T-cell repertoire has been shown to shrink dramatically due to the progressive depletion of naive T cells in the peripheral reserve pool. It is this shortage of responsive naive T cells that has been thought to be responsible for the susceptibility of elderly people to infection, cancer, and poor outcomes following vaccination [20]. Various interventions, including caloric restriction [7], exercise [21], and vitamin E supplementation [8], have all been used to successfully replenish naive T cells and thereby extend life span and reduce onset of infection and cancer among the elderly. In this study, we have demonstrated that ECD is able to increase the level of naive T cells in the peripheral pool and there was a concomitant extension of life span and reduction in the frequency of tumor formation in senescence-accelerated mice (ECD therapeutic group versus SAM-P8, 0 versus 1/6). Therefore, it seems reasonable to conclude that ECD's capacity to reverse the age-dependent depletion of peripheral naive T cells may contribute to its known effects in reducing age-related disease which derive from long-term clinical observations.

In the normal immune response, following the containment and elimination of a foreign antigen, most of the clonally expanded T cells undergo apoptosis with only a small fraction being retained as memory T cells. Consequently, as an immune-competent organism ages, peripheral memory T cells accumulate as a result of a lifetime of exposure to foreign antigens converting naive T cells into memory cells [22, 23]. A number of studies have suggested that defects of lymphocyte apoptosis in older animals and humans also contribute to enlargement of the memory T cell compartment [24, 25]. In addition a recent study has shown that an overabundance of peripheral memory T cells can inhibit homeostatic proliferation of naive T cells [26]. These recent studies have also suggested that memory T cells may secrete high levels of proinflammatory cytokines which may contribute to persistent inflammations in various tissues of the elderly. Consequently reducing the level of memory T cells should be beneficial both for homeostatic proliferation of naive T cells and also in alleviating the excess inflammatory state seen in older organisms. In this study, dietary supplementation with ECD was shown to be able to reduce the level of peripheral memory T cells and increase levels of naive T cells. In addition, ECD supplementation increased apoptosis in peripheral lymphocyte populations, potentially making space for new naive lymphocytes. Necrosis in peripheral lymphocytes, which is mainly induced by oxidative stress in senile animals, was also inhibited.

Extensive evidence exists to indicate that aging in an organism is not only characterized with immune deficiency, but also by an increase in chronic inflammation [27]. Proinflammatory status is most clearly manifested in increased levels of proinflammatory cytokines, including IL-6, TNF-*α*, and IL-1*β*, in the circulation coupled with increased frequencies of chronic inflammatory diseases associated with aging such as Alzheimer's disease, Parkinson's diseases, and atherosclerosis. One of the most prominent inflammatory cytokines is IL-6, and its plasma concentration increases with aging and age-related diseases [18, 28, 29] and epidemiological studies suggest IL-6 is a good biomarker of longevity as it is closely related to mortality in elderly patient cohorts [30, 31]. Consistent with this, a number of therapeutic interventions that decrease the IL-6 concentration are able to alleviate age-related diseases [18]. Against this background, it was significant that in this study dietary supplementation with ECD was able to reduce peripheral IL-6 concentrations, with the implication that this effect may contribute to the underlying mechanisms of reducing frequency of age-related diseases that have been obtained through long-term use of ECD in clinical practice.

Alongside the changes in the adaptive immune response that occur with age, there are also major changes in innate immunity as an organism ages. Amongst these, changes in natural killer [18] cells, the first line of cellular components to provide direct cytotoxic lysis of tumor cells and virus infected cells, have been extensive since the elderly people show increased susceptibility to both tumor development and virus infections [32]. This study indicated that CD3+Ly49+ NK cells were present at significantly lower levels in senile SAM-P8 mice compared to SAM-R1 control animals, which may be another underlying factor accounting for the increased susceptibility of elderly mice to cancer and virus infection. The increase in the level of NK cells seen in this study following dietary supplementation with ECD could therefore be beneficial to elderly organisms in terms of increased resistance to tumor development and virus infection. However, further studies are required to clarify the role of the effect of ECD on NK cells in aged organisms.

In conclusion, this study has shown that treatment of the senescence-accelerated mouse prone substrain, SAM-P8, with ECD can induce a significant reversal of age-related immunosenescence alterations. Deficiencies in both peripheral and spleen cell populations of naive T cells and NK cells were reduced, while levels of redundant memory T cells were also reduced. In addition, dietary supplementation with ECD was able to suppress both necrosis in peripheral lymphocytes and the levels of the proinflammatory cytokine, IL-6. Finally in addition to bringing about changes in the senile immune system, ECD dietary supplementation significantly prolonged the life span of senile SAM-P8 mice.

## Figures and Tables

**Table 1:** The major components in extracts of *Cistanche deserticola*.

Sample	Echinacoside (% w/w)	Acteoside (% w/w)	8-Epiloganic acid (% w/w)	Oligosaccharides (% w/w)
ECD	8.25	3.80	5.89	82.01
